# Phenotypic features of chronic migraine

**DOI:** 10.1186/s10194-016-0616-y

**Published:** 2016-03-15

**Authors:** Osman Özgür Yalın, Derya Uluduz, Aynur Özge, Mehmet Ali Sungur, Macit Selekler, Aksel Siva

**Affiliations:** Neurology Department, Istanbul Education and Research Hospital, Kasap İlyas Mah. Org. Abdurrahman, Nafiz Gürman Cd, PK: 34098 Fatih, Istanbul, Turkey; Neurology Department, Istanbul University Cerrahpasa School of Medicine, İstanbul, Turkey; Neurology Department, Mersin University School of Medicine, Mersin, Turkey; Biostatistics Department, Düzce University School of Medicine, Düzce, Turkey; Neurology Department, Kocaeli University School of Medicine, Kocaeli, Turkey

**Keywords:** Chronic migraine, Phenotype, Accompanying symptoms, Chronification

## Abstract

**Background:**

Chronic migraine is a disabling, under-recognized, and undertreated disorder that increases health burdens. The aim of this study was to evaluate phenotypic features and the relevance of accompanying symptoms of migraine attacks in chronic migraine.

**Method:**

This study was conducted as part of an ongoing Turkish Headache Database Study investigating the clinical characteristics and outcomes of headache syndromes in the Turkish population. The electronic database was examined retrospectively, and 835 patients with chronic migraine were included.

**Results:**

Patient group consisted of 710 women and 125 men (85 and 15 %, respectively). Mean patient age was 36.8 ± 13.5 years, median value of migraine onset was 60 months (18–120), median headache frequency was 25 days per month (16–30), median of attack duration was 12 h (4–24), and median of intensity was eight (7–9). Increasing headache days per month were inversely related with the presence of nausea, vomiting, phonophobia, and photophobia. Longer duration of headache (months) and higher visual analog scale (VAS) for headache intensity were associated with all accompanying symptoms. Phonophobia, nausea, photophobia, and vomiting were the most frequent accompanying symptoms (experienced by 80.2, 77.6, 71.2, and 40.9 % of patients, respectively). Osmophobia was also frequent in chronic migraine patients (53.4 %) and was closely associated with other accompanying symptoms. Vertigo and dizziness were observed less frequently, and they were not associated with accompanying symptoms.

**Conclusion:**

Phenotype of chronic migraine may be associated with the course of chronification. Duration of illness and attack intensity were closely related with the presence of accompanying symptoms, although headache frequency was found to be inversely related to the presence of accompanying symptoms. Osmophobia was also a frequent symptom and was closely related with other accompanied symptoms, unlike vertigo and dizziness. Inclusion of osmophobia into the diagnostic criteria might improve accurate diagnosis of chronic migraine.

## Background

Chronic migraine (CM) is defined by the presence of headache attacks on 15 days or more per month for at least three months of which 8 or more days meet criteria for migraine [1]. A prevalence rate of 2 % for CM has been estimated [2–4]. Although CM is much less common than episodic migraine (EM), it is substantially more debilitating, with a greater negative effect on quality of life, and it is a challenging disorder for practitioners [4, 5]. Despite the substantial personal and social consequences of CM, its diagnostic criteria are still evolving [6]. The third edition of the International Classification of Headache Disorders (ICHD-3 beta) [1], published in 2013, has comprehensively described primary chronic headache disorders, such as CM, chronic tension-type headache, new daily persistent headache, and chronic hemicrania continua [1]. In this new edition, medication overuse headache is not an exclusion criterion; therefore, CM now represents a broader patient group.

CM is a disabling, underdiagnosed, and undertreated disorder [6]. CM patients constitute the most debilitated subgroup of the migraine population, and may therefore represent a clinically distinct group [7]. According to the American Migraine Prevalence and Prevention study, patients with CM were twice as likely to develop depression, anxiety, and chronic pain than those with EM [8]. Cutaneous allodynia, anxiety, disability, and depression are frequently considered as predictors of chronification [9, 10].

The explicit mechanisms of CM are not fully understood. Atypical modulation of pain may play a role in its pathogenesis [11]. Pain modulatory pathway alterations have been identified in CM [12]. A suggested mechanism for chronification of migraine is that repeated migraine attacks could have cumulative structural effects on the trigeminovascular pathway [13]. This theory is supported by the evidence of iron deposition in the periaqueductal gray matter of patients with a long history of migraine [14]. In addition, CM is associated with a greater degree of impairment in cortical processing of sensory stimuli [15]. Therefore, proposed structural changes in the excitability of the trigeminovascular pathway and/or other brainstem pain modulating systems could result in different phenotypes of the disease.

According to the ICHD-3 beta diagnostic criteria of migraine, accompanying symptoms item (D) require either (1) nausea and/or vomiting and/or (2) photophobia and phonophobia. Although osmophobia is mentioned as an accompanying symptom in the appendix-definitions section, it has not been included in the diagnostic criteria. Osmophobia refers to an increased sensitivity to odors during migraine attacks, and it seems to be a distinguishing feature of migraine [16]. Association between osmophobia and allodynia has also been investigated recently, and it is also proposed as a shared central sensitization mechanism [17].

To the best of our knowledge, the association between headache characteristics and accompanying symptoms of CM has not been reported yet. The aims of this study are (1) to determine the clinical characteristics of headache attacks and to investigate the relevance of associated symptoms such as nausea, vomiting, phonophobia, and photophobia; (2) to investigate other associated symptoms that could improve diagnostic certainty, including osmophobia, dizziness, and vertigo; and (3) to analyze relationships among associated symptoms.

### Methods

This retrospective observational study was a part of ongoing Turkish Headache Database (THD) Study. THD investigating the clinical characteristics and outcomes of primary and secondary headache syndromes in Turkish population is supported by Turkish Neurological Society. Out of tertiary headache centers in THD, patients diagnosed with CM based on ICHD-2 criteria between the years 2008 and 2012 in Mersin University, Istanbul University and Kocaeli University were enrolled to this study. The Human Ethical Committee of Mersin University (MEU. 0.01.00.06/265, 20.10.2008) approved this non-profit study. Subjects meeting the inclusion criteria were enrolled into the study after giving written consent. Diagnosis of migraine was done by headache experts with face-to-face examinations according to ICHD-2 criteria [18]. Subjects younger than 18 years of age and those older than 80 years of age (*n* = 123 subjects), those who had received a headache diagnosis other than CM (*n* = 2850 episodic migraine subjects), and patients with chronic TTH, hemicrania continua, medication overuse headache and patients who had TTH attacks more frequently than migraine attacks (*n* = 385 subjects) were excluded. Data on socio-demographic and clinical characteristics, including age at onset, headache duration, headache frequency, presence of aura, headache characteristics, localization, intensity of pain, triggering factors, associated features, comorbidity, and medical and family history were gathered from a web-based database. Accompanying symptoms of CM were evaluated based on ICHD-2 criteria. Presence of vertigo, dizziness and osmophobia were also analyzed retrospectively taking into consideration of ICHD-3 beta criteria. Vertigo and dizziness is stated when related with migraine attacks. Vertigo definition based to clinical features: internal/external vertigo, positional, occurring during head motion. Similarly dizziness defined sensation of disequilibrium occurring during headache attack, discrimination is particularly based to clinical examination of experts.

When extracting data from the electronic database, we excluded all missing data, including missing data on accompanying symptoms. Therefore, we obtained a reduced number of subjects in osmophobia assessments. Although this could limit the power of the analysis, it also increased the reliability of the data and prevented errors that could be attributed to the retrospective design of the study. The visual analog scale (VAS) was used for assessment of pain intensity.

### Statistical analysis

Data were entered and analyzed in the statistical analysis program PASW v.18 (SPSS Inc. Released 2009, PASW Statistics for Windows, Version 18.0. Chicago: SPSS Inc.) statistical package. Distributions of data were examined with the Shapiro-Wilk test, and independent samples t test and one-way ANOVA or non-parametric alternatives (Mann–Whitney U and Kruskal-Wallis tests) were used to compare groups according to the distribution of dependent variables. Continuous variables were summarized as mean ± standard deviation or median (25–75 %) according to the distribution, and categorical variables were summarized as frequency and percentile. Spearman non-parametric correlations were used to analyze relationships among headache properties. Pearson-Chi square or Fisher’s exact test were used according to the expected count rule for categorical data. Two-tailed *p* < 0.05 was accepted as significant.

## Results

The study group consisted of 835 CM patients, 710 female (85 %), mean age 36.8 ± 13.5 years. The median value of migraine onset was 60 months (18–120), median headache duration was 12 h (4–24) and median headache intensity was eight (7–9). Seventy-seven percent of CM patients described throbbing headache followed by pressing (13.7 %) and dull (5.5 %) headache. The localization of headache was predominantly unilateral (39.5 %), followed by bifrontal (10.7 %), generalized (14.8 %), and sub-occipital (13.6 %). Phonophobia (80.2 %) was the most common accompanying symptom, followed by nausea (77.6 %) and photophobia (71.2 %). Vomiting was present in 40.9 % of patients. Osmophobia was observed in 53.4 % of patients, and dizziness and vertigo was present in 17.5 % and 14.4 % of patients respectively. Characteristics of study patients is summarized at Table 1. The most common triggering factor was emotional stress (79.2 %), followed by skipping meals (57.0 %), menstruation (36.7 %), and seasonal factors (23.0 %). Most of the patients reported no intraday-difference, but for 31.4 % of patients, headaches were most intense in the evening. Headache frequency did not change with age. Headache frequency was inversely related with headache onset (*r* = -0.131, *p* < 0.001), attack duration (*r* = -0.110, *p* = 0.002), and intensity of headache (*r* = -0.118, *p* = 0.002). When correlation analysis adjusted according to gender and age covariates; correlation of headache onset disappeared, headache frequency was inversely related with attack duration (*r* = -0.083, *p* = 0.032), and intensity of headache (*r* = -0.139, *p* < 0.001). Analysis of gender effects revealed no difference between males and females in terms of headache frequency (*p* = 0.589), duration of illness (*p* = 0.054), or intensity (*p* = 0.559). Attack duration (hours) was significantly longer for females (*p* < 0.001) (Table 2).

**Table 1:** Headache characteristics of study group

Gender	Female	710 (85 %)
	Male	125 (15 %)
Age	Age	36,8 ± 13,5
Headache characteristics	Duration of illness (months)	60 (18–120)
	Headache frequency (days/month)	25 (16–30)
	Headache duration (hours)	12 (4–24)
	Headache intensity (VAS)	8 (7–9)
Quality of headache (*n* = 817)	Throbbing headache	631 (77.2 %)
	Pressing	112 (13.7 %)
	Dull	45 (5.5 %)
	Others	29 (3.5 %)
Headache localization (*n* = 811)	Unilateral	320 (39.5 %)
	Bifrontal	87 (10.7 %)
	Generalized	120 (14.8 %)
	Sub-occipital	110 (13.6 %)
	Others	198 (21.4 %)
Accompanying symptoms	Phonophobia (*n* = 792)	635 (80.2 %)
	Nausea (*n* = 789)	612 (77.6 %)
	Photophobia (n = 787)	560 (71.2 %)
	Vomiting (*n* = 738)	302 (40.9 %)
	Osmophobia (*n* = 238)	127 (53.4 %)
	Dizziness (*n* = 661)	116 (17.5 %)
	Vertigo (*n* = 641)	92 (14.4 %)

*VAS* visual analog scale

**Table 2:** Headache characteristics according to gender

	Male (*n* = 125)	Female (*n* = 710)	*p*
Headache frequency (days/month)	20 [16–30]	25 [16–30]	0.589
Duration of illness (months)	48 [12–120]	60 [24–132]	0.054
Headache duration (hours)	7 [2–24]	12 [4–24]	<0.001
Headache intensity (VAS)	8 [7–9]	8 [7–9]	0.559

*VAS* visual analog scale

To reveal the phenotypic features of CM, we compared headache frequency, duration of attack, intensity, and duration of illness according to the presence of accompanying symptoms. First, we compared headache frequency (headache days per month) in terms of the presence of accompanying symptoms. We observed that the presence of nausea, vomiting, phonophobia, and photophobia were significantly associated with fewer headache days per month (Table 3). When we investigated attack duration, we observed that vomiting, phonophobia, and photophobia were associated with longer duration of attack (Table 4). Greater headache intensity (VAS) and longer duration of illness (in months) were significantly associated with the existence of all accompanying symptoms, including nausea, vomiting, phonophobia, photophobia, dizziness, vertigo, and osmophobia (see Tables 5 and 6 for intensity and duration, respectively).

**Table 3:** Headache frequency (days/month) according to accompanying symptoms

Accompanying symptoms		Number of subjects (*n*)	Headache days/month	*P* value
Nausea	Present	612	20 [15–30]	*0.008*
	Absent	177	30 [16–30]	
Vomiting	Present	302	20 [15–30]	*<0.001*
	Absent	436	30 [18–30]	
Phonophobia	Present	635	20 [15–30]	*0.002*
	Absent	157	30 [20–30]	
Photophobia	Present	560	20 [15–30]	*<0.001*
	Absent	227	30 [20–30]	
Dizziness	Present	116	25 [16–30]	0.056
	Absent	545	20 [16–30]	
Vertigo	Present	92	25 [16–30]	0.440
	Absent	549	24 [16–30]	
Osmophobia	Present	127	20 [16–30]	0.371
	Absent	111	20 [15–30]

**Table 4:** Headache duration (hours) according to accompanying symptoms

Accompanying symptoms		Number of subjects (*n*)	Headache duration (hours)	*P* value
Nausea	Present	612	12 [4–24]	0.056
	Absent	177	12 [3–24]	
Vomiting	Present	302	19 [6–24]	*<0.001*
	Absent	436	12 [3–24]	
Phonophobia	Present	635	12 [4–24]	*0.010*
	Absent	157	11 [4–24]	
Photophobia	Present	560	12 [4–24]	*0.003*
	Absent	227	10 [3–24]	
Dizziness	Present	116	12 [6–24]	0.148
	Absent	545	12 [4–24]	
Vertigo	Present	92	12 [5–24]	0.826
	Absent	549	12 [4–24]	
Osmophobia	Present	127	12 [7–24]	0.219
	Absent	111	12 [4–24]

**Table 5:** Headache intensity (VAS) according to accompanying symptoms

Accompanying symptoms		Number of subjects (*n*)	Headache intensity (VAS)	*P* value
Nausea	Present	612	8 [7–10]	*<0.001*
	Absent	177	7 [6–9]	
Vomiting	Present	302	8 [7–10]	*<0.001*
	Absent	436	7 [6–9]	
Phonophobia	Present	635	8 [7–10]	*<0.010*
	Absent	157	7 [6–8]	
Photophobia	Present	560	8 [7–10]	*<0.001*
	Absent	227	7 [6–8]	
Dizziness	Present	116	8 [7–10]	*0.004*
	Absent	545	8 [7–9]	
Vertigo	Present	92	8 [7–10]	*0.006*
	Absent	549	8 [7–9]	
Osmophobia	Present	127	8 [7–10]	*0.032*
	Absent	111	8 [7–9]

**Table 6:** Duration of illness (months) according to accompanying symptoms

Accompanying symptoms		Number of subjects (*n*)	Duration of illness (months)	*P* value
Nausea	Present	612	60 [24–156]	*<0.001*
	Absent	177	36 [10–114]	
Vomiting	Present	302	108 [36–180]	*<0.001*
	Absent	436	36 [12–120]	
Phonophobia	Present	635	60 [24–156]	*<0.001*
	Absent	157	36 [9–96]	
Photophobia	Present	560	72 [24–166]	*<0.001*
	Absent	227	36 [12–96]	
Dizziness	Present	116	114 [36–240]	*<0.001*
	Absent	545	48 [12–120]	
Vertigo	Present	92	82 [24–180]	*0.015*
	Absent	549	48 [12–120]	
Osmophobia	Present	127	120 [48–240]	*<0.001*
	Absent	111	48 [12–120]	

We also analyzed the associations of accompanying symptoms with each other (Table 7). The diagnostic accompanying symptoms described in ICHD-2 and also in ICHD-3 beta are nausea, vomiting, phonophobia, and photophobia, and these symptoms were found to be closely related with each other (*p* = 0.001). The crosstab analysis revealed that osmophobia and dizziness were significantly correlated with the presence of other diagnostic accompanying symptoms. By using *x*^2^ tests, we found that osmophobia was associated with more frequent incidences of nausea (*p* = 0.006), vomiting (*p* = 0.013), phonophobia (*p* = 0.001), and photophobia (*p* = 0.001). Dizziness was associated with more frequent incidences of nausea (*p* = 0.008), vomiting (0.001), phonophobia (*p* = 0.001), and photophobia (*p* = 0.001). The presence of vertigo was not associated with nausea (*p* = 0.445), vomiting (*p* = 0.142), or photophobia (*p* = 0.063). A detailed analysis of the accompanying symptoms is presented in Table 7.

**Table 7:** Associations of accompanying symptoms with each other

		Nausea	Vomiting	Phonophobia	Photophobia	Dizziness	Vertigo	Osmophobia
Nausea	+		51.2	86.1	77.4	19.7	14.9	57.8
	-		7.9	58.0	47.4	10.6	12.4	35.4
	*p*		*0.001*	*0.001*	*0.001*	*0.008*	0.445	*0.006*
Vomiting	+	95.3		88.2	83.1	25.0	16.5	59.8
	-	62.6		71.9	59.4	12.6	12.4	43.1
	*p*	*0.001*		*0.001*	*0.001*	*0.001*	0.142	*0.013*
Phonophobia	+	83.5	45.5		82.1	20.4	16.8	59.6
	-	52.9	22.3		27.4	6.6	6.6	19.5
	*p*	*0.001*	*0.001*		*0.001*	*0.001*	*0.002*	*0.001*
Photophobia	+	84.7	48.8	92.3		21.2	16.0	59.7
	-	59.3	22.1	49.6		9.3	10.5	28.0
	*p*	*0.001*	*0.001*	*0.001*		*0.001*	0.063	*0.001*
Dizziness	+	85.2	55.3	91.2	85.2		50.0	71.3
	-	73.5	34.7	73.9	64.0		6.2	32.0
	*p*	*0.008*	*0.001*	*0.001*	*0.001*		*0.001*	*0.001*
Vertigo	+	78.0	44.9	89.1	75.8	59.3		65.7
	-	74.3	36.8	74.3	65.9	8.8		39.9
	*p*	0.445	0.142	*0.002*	0.063	*0.001*		*0.001*
Osmophobia	+	86.4	53.5	93.5	88.8	60.8	43.6	
	-	71.8	36.9	70.3	67.6	22.7	21.1	
	*p*	*0.006*	*0.013*	*0.001*	*0.001*	*0.001*	*0.001*	

In this table, + lines indicate subjects who had related symptoms, and – lines indicate subjects who did not have related symptoms

## Discussion

Our study revealed that the presence of diagnostic accompanying symptoms (nausea, vomiting, phonophobia, and photophobia) lessened with higher numbers of headache days per month. This finding is compatible with the literature. Migraine characteristics, especially accompanying symptoms, are less prominent in the CM population [19]. This could be important for two reasons: first the absence of accompanying symptoms could lead to underdiagnosis of CM. Second, this observation could point to cumulative effects of frequent headaches. CM is described as an altered entity, and there is growing evidence of chronicity-related structural effects in pain pathways [20]. Interestingly, in our population, headache frequency did not change with age or gender. Duration of illness and intensity scores (VAS) were also similar for both genders. However, headache duration was significantly longer in females. Slater et al. reported longer headache duration in females in childhood migraine [21]. Adult population studies also report longer headache duration in females [22, 23], suggesting a female gender tendency in CM.

Both duration of illness (months) and headache intensity (VAS) scores were found to be significantly associated with the presence of all accompanying symptoms, including nausea, vomiting, phonophobia, photophobia, dizziness, vertigo, and osmophobia. Although our study covered only CM patients, our results indicate that duration of illness and headache intensity are closely related with accompanying symptoms. Kelman et al. reported that headache intensity was correlated with nausea, vomiting, photophobia, phonophobia, and dizziness, and headache duration was correlated with osmophobia and taste abnormalities [23]. In our results, accompanying symptoms were present as hallmarks of duration of illness and attack intensity in the CM population.

Attack duration was found to be related to the presence of vomiting, phonophobia, and photophobia. However, the presence of other accompanying symptoms—including nausea, osmophobia, vertigo, and dizziness—did not change with varying attack duration. Conversely, Kelman and Tanis reported a correlation between attack duration and osmophobia and taste abnormalities. However, their study also covered EM patients [23]. This difference could point to the effects of chronification on attack phenotype.

Finally, we analyzed the relationships of associated symptoms with each other. The results revealed that nausea, vomiting, phonophobia, and photophobia are closely correlated with each other. Osmophobia (53.4 %) was also a common accompanying symptom. In addition, the presence of osmophobia was significantly associated with other diagnostic accompanying symptoms. Because ICHD-3 beta did not include other common symptoms accompanying diagnostic criteria, there is insufficient data investigating the predictive value of such symptoms. The diagnostic utility of osmophobia was proposed previously by some authors, especially for Asian migraineurs, in whom photophobia is less common [16]. Similarly, Silva-Neto et al. reported that osmophobia may be a specific marker for migraine and suggested its inclusion within the diagnostic criteria [24]. According to a recent study, the prevalence of both allodynia and osmophobia were higher in chronic than in episodic migraineurs [17]. Same study also reported a relationship between duration of headache and osmophobia.

This study has some limitations. First, the retrospective study design limited our data to monitoring blood pressure, body mass index, and educational-socioeconomic status. Second, this study did not include EM patients, so we could not compare our findings with those of EM sufferers. Due to the retrospective design of this study some missing values of the parameters was one of our limitations. But even so power analysis of the data is mostly estimated 80–99 % for variables. However, this study gives us clues about the determinants of phenotype in CM populations.

## Conclusion

In conclusion, our study supports and expands the literature and could be summarized as follows: (1) CM is defined as 15–30 headache days per month, and in our study population, higher numbers of headache days were found to be correlated with less prominent nausea, vomiting, photophobia, and phonophobia; (2) long duration of headache (months) and higher attack intensity (VAS) were found to be correlated with more frequent incidences of all accompanying symptoms investigated; (3) longer attack duration (hours) was found to be correlated with the presence of vomiting and phono-photophobia; (4) duration of headache and attack intensity are the most relevant determinants of CM attack phenotype; (5) osmophobia is also common in CM and is associated with diagnostic accompanying symptoms; and (6) dizziness and vertigo were less frequent in the CM population and do not have a prominent relationship with other diagnostic accompanying symptoms.

Future studies focusing on clinical presentation and accompanying symptoms could provide additional data to shed light on the phenotypes, classifications, and pathophysiological pathways of chronicity.

## Abbreviations

CM: chronic migraine
ICHD-3 beta: the third edition of the International Classification of Headache Disorders
VAS: visual analogue scale
